# Low plasma levels of BTLA and LAG-3 before HCV therapy are associated with metabolic disorders after HCV eradication in persons with HIV/HCV coinfection: a retrospective study

**DOI:** 10.3389/fphar.2024.1341612

**Published:** 2024-10-28

**Authors:** Rubén Martín-Escolano, Ana Virseda-Berdices, Juan Berenguer, Juan González-García, Oscar Brochado-Kith, Amanda Fernández-Rodríguez, Cristina Díez, Victor Hontañon, The Marathon Study Group, A. Arranz, Salvador Resino, María Ángeles Jiménez-Sousa

**Affiliations:** ^1^ Centro Nacional de Microbiología (CNM), Unidad de Infección Viral e Inmunidad, Instituto de Salud Carlos III (ISCIII), Madrid, Spain; ^2^ Centro de Investigación Biomédica en Red en Enfermedades Infecciosas (CIBERINFEC), Instituto de Salud Carlos III (ISCIII), Madrid, Spain; ^3^ Unidad de Enfermedades Infecciosas/VIH, Hospital General Universitario “Gregorio Marañón”, Madrid, Spain; ^4^ Instituto de Investigación Sanitaria Gregorio Marañón (IiSGM), Madrid, Spain; ^5^ Servicio de Medicina Interna-Unidad de VIH, Hospital Universitario La Paz, Madrid, Spain; ^6^ Instituto de Investigación Sanitaria La Paz (IdiPAZ), Madrid, Spain

**Keywords:** HIV/HCV-coinfection, HCV therapy, immune checkpoint proteins, type 2 diabetes mellitus, dyslipidemia, TyG index

## Abstract

**Background:**

Understanding the predictors of metabolic disorders in persons with HIV/HCV coinfection post-HCV therapy is crucial for improving patient outcomes. Since immune checkpoint proteins are usually upregulated in these persons with HIV/HCV coinfection, we aimed to evaluate the association between plasma immune checkpoint proteins at baseline (before HCV therapy) and metabolic disturbances during the follow-up (about 5 years after successful HCV treatment) in persons with HIV/HCV coinfection.

**Methods:**

We performed a retrospective study on 80 persons with HIV/HCV coinfection with advanced fibrosis or cirrhosis who cleared HCV infection after successful HCV therapy and were followed for about 5 years after completion of HCV treatment. Plasma samples were collected at baseline. Immune checkpoint proteins were analyzed using a Luminex 200™ analyzer. Outcomes were the development of a metabolic event (type 2 diabetes mellitus and/or dyslipidemia) and the change in Triglycerides and glucose (TyG) index.

**Results:**

During follow-up, 21 (26%) patients developed metabolic events (type 2 diabetes mellitus/dyslipidemia), and 29 (46.0%) patients had an increase in TyG during the follow-up. Low baseline values of BTLA and LAG-3, two immune checkpoint proteins, were associated with the development of metabolic events (aAMR = 0.69 and aAMR = 0.71, respectively) and with increases in TyG values (aAMR = 0.72 and aAMR = 0.70, respectively). In addition, other immune checkpoint proteins were also inversely associated with increases in TyG.

**Conclusion:**

We discovered that low plasma levels of BTLA and LAG-3 before HCV therapy significantly correlate with an increased risk of developing metabolic disorders after treatment.

## Introduction

Chronic hepatitis C virus (HCV) infection causes several complications related to metabolic alterations, such as dyslipidemia, insulin resistance, and type 2 diabetes mellitus (TD2M), among others (Spearman et al., 2019). Coinfection with human immunodeficiency virus (HIV) increases HCV viremia and the incidence of newly diagnosed cases of disease (Chen et al., 2014), leading to more rapid progression of HCV-associated hepatic disease than HCV monoinfected patients (Macias et al., 2012). In addition, persons with HIV/HCV coinfection have an increased risk of metabolic alterations, mediated by patient-specific factors, viral-mediated effects, and antiretrovial therapy (ART) exposure (Collins et al., 2019).

The impact of HCV eradication after successful therapy on the evolution of liver and non-liver complications related to HCV infection has been extensively debated (Rockey and Friedman, 2021). In this sense, persistent molecular changes associated with the risk of severe disease and caused by chronic hepatitis C could explain that HCV cure only partially reduces this risk (Perez et al., 2019). Metabolic alterations, one of the hallmarks of chronic hepatitis C, may persist after HCV clearance and further drive steatosis, nonalcoholic fatty liver disease (NAFLD), and liver disease progression (Fouad et al., 2021), as well as increase the risk of cardiovascular events, cancer, and mortality (Wang et al., 2020).

HIV and HCV infections dysregulate immunity, generating a low-grade chronic inflammatory state that accompanies the metabolic disturbances in patients infected with HCV and/or HIV (Mazzuti et al., 2023; Nevola et al., 2021). Immune checkpoint proteins (ICPs) are regulatory molecules that maintain balance within the immune system (Gaikwad et al., 2022). Several ICPs are dysregulated in hepatitis C, leading to the persistence and pathogenesis of HCV infection, and cannot be restored entirely after HCV clearance (Martin-Escolano et al., 2023; Chen et al., 2022). In HIV infection, these ICPs are elevated in persons who were ART-naïve, decrease after ART, but remain elevated compared to healthy people (Chew et al., 2016), regardless of whether treatment is started early or late after HIV infection (Rutishauser et al., 2017), and correlated with disease progression as reflected in both decreased T cell function and CD4^+^ T cell counts, as well as increased viral RNA replication and HIV reservoir (Sun and Xue, 2022). Regarding HIV/HCV coinfection, several IPCs such as CD27, TIM-3, PD-1 or IDO have also been found to be elevated (Shata et al., 2013; Caraballo Cortes et al., 2023; Hoel et al., 2020; Polo et al., 2019; Barrett et al., 2015). These IPCs decrease after both ART and/or DAAs treatment initiation (Caraballo Cortes et al., 2023; Hoel et al., 2020; Farcomeni et al., 2021), but also remain elevated compared to healthy people.

Some ICPs have been related to metabolic disorders, such as programmed cell death protein 1(PD-1) and programmed death-ligand 1 (PD-L1) with TD2M and obesity (Yang et al., 2022). However, while previous studies have focused on the relationship between immune checkpoint proteins and liver disease outcomes, the role of these proteins in post-therapy metabolic disorders in persons with HIV/HCV coinfection remains unexplored. In this regard, HCV is considered a virus linked to metabolic disturbances, and some patients achieving sustained virologic response (SVR) continue at risk of developing metabolic syndrome (Chaudhari et al., 2021). Therefore, in the era of curative HCV therapy based on direct antiviral agents (DAAs), the search for predictive biomarkers of disease is necessary to more closely monitor patients who remain at risk of developing long-term liver and non-liver events. Besides, it is important to note that the potential risk of developing metabolic disorders is higher in the presence of advanced fibrosis or cirrhosis (Zein et al., 2000; Lee et al., 2019; Kobashi-Margain et al., 2010; Wlazlo et al., 2010; Unger et al., 2019). Therefore, it is vital to investigate the pathophysiological mechanisms involved in liver disease progression, as well as the development of metabolic disorders among patients with hepatitis C and advanced fibrosis or cirrhosis who have cleared HCV infection.

In this work, we aimed to evaluate the association between plasma levels of ICPs at baseline (before HCV therapy) and the development of metabolic disturbances about 5 years after successful HCV treatment in persons with HIV/HCV coinfection and advanced fibrosis or cirrhosis.

## Materials and methods

### Study subjects

We performed a multicenter retrospective study between 2012 and 2021 on 80 persons with HIV/HCV coinfection who were cleared of HCV infection after HCV interferon (IFN)-based therapy (peg-IFN-α/ribavirin or peg-IFN-α/ribavirin/DAAs) or IFN-free DAAs therapy. All patients were collected from the GeSIDA 10318 cohort/Marathon study, which had the following inclusion criteria: i) advanced fibrosis or cirrhosis, ii) a stable ART for over 6 months and an undetectable plasma HIV viral load (<50 copies/mL) ii) SVR achievement (undetectable HCV-RNA load 12–24 weeks – depending on regimen – after the finalization of anti-HCV treatment), iii) available frozen plasma samples before HCV therapy (baseline) and clinical data at baseline and during the follow-up (median of 5 years after finishing HCV treatment). The end of follow-up was between 2019 and 2021. Patients with hepatitis B virus (HBV) coinfection, acute hepatitis C, or previous metabolic events (diabetes mellitus and dyslipidemia) were excluded.

The study was approved by the Research Ethics Committee of the Institute of Health Carlos III (CEI PI 72_2021) and conducted following the Declaration of Helsinki. All participants signed a written consent to participate in the study.

For each patient, clinical and epidemiological data were collected from the medical records using an online form, information that was treated with confidentiality and monitored. Besides, a peripheral blood sample was collected at baseline (before HCV therapy) in EDTA tubes by venipuncture and sent to the HIV BioBank. Plasma aliquots were obtained by centrifugation and were stored frozen (−80°C) until use.

### Multiplex immunoassays

Immuno-Oncology Checkpoint 14-Plex Human ProcartaPlex™ Panel 1 (Invitrogen™) in a Luminex 200™ analyzer (Luminex Corporation, Austin, TX, United States) was used to measure several plasma ICPs. Luminex 200TM analyzer allows the simultaneous detection and quantification of a large number of secreted proteins in a single well, being a valid, cost- and time-effective alternative to the classic ELISA that can only measure a single protein at a time.

The panel includes ICPs that play a crucial role in the regulation of T cells, leading to either T cell exhaustion [B and T lymphocyte attenuator (BTLA), cluster of differentiation 80 (CD80), CD152(CTLA4), indoleamine 2,3-dioxygenase (IDO), lymphocyte activation gene-3 (LAG-3), programmed cell death protein 1(PD-1), programmed death-ligand 1 (PD-L1), programmed death-ligand 2 (PD-L2), and T-cell immunoglobulin and mucin-domain containing-3 (TIM-3)] or stimulation [CD27, CD28, CD137(4-1BB), glucocorticoid-induced TNFR-related (GITR), and herpesvirus entry mediator (HVEM)]. The measured raw fluorescence intensity (FI) values (arbitrary units, a.u.) were used.

### Outcome variables

Diabetes mellitus and dyslipidemia developed during the follow-up (about 5 years after completing HCV treatment) were considered primary outcomes (dichotomous). Diabetes mellitus was defined as symptoms of diabetes (polyuria, polydipsia, unexplained weight loss, as defined by the 2003 Expert Committee on Diabetes Mellitus) plus casual plasma glucose concentration 200 mg/dL, or fasting plasma glucose concentration ≥126 mg/dL, or 2 h plasma glucose ≥200 mg/dL during an oral glucose tolerance test (D Expert Committee on the and and M Classification of Diabetes, 2003). Dyslipidemia was defined as total cholesterol ≥200 mg/dL, low-density lipoprotein (LDL) cholesterol ≥130 mg/dL, or serum triglycerides ≥150 mg/d (E Expert Panel on Detection and and A Treatment of High Blood Cholesterol in, 2001). Diagnosis criteria for both diabetes mellitus and dyslipidemia were consistent at baseline and follow-up.

The change in the Triglycerides and Glucose (TyG) index between the beginning and the end of follow-up, coding dichotomously (∆TyG>0 versus ∆TyG <0), was a secondary outcome. The TyG index was calculated using the following formula: TyG = ln [fasting triglyceride (mg/dL) × fasting plasma glucose (mg/dL)/2] (Nabipoorashrafi et al., 2022).

### Statistical analysis

For descriptive studies, quantitative variables (clinical and epidemiological variables) were expressed as median (interquartile range, IQR), and categorical variables were shown as absolute count (percentage). Independent groups were compared using the Mann-Whitney U and Chi-square tests for quantitative and categorical variables, respectively. Dependent groups were compared using the Wilcoxon signed range test for continuous variables.

A generalized Linear Model (GLM) with gamma distribution (log-link) was used to analyze the association between plasma ICPs at baseline and the dichotomous outcome variables, providing the arithmetic mean ratio (AMR), the 95% of confidence interval (95% CI), and its level of significance. All GLMs were adjusted for available patient characteristics (age, gender, body mass index (BMI), total cholesterol, and HCV viral load). Baseline LDL cholesterol was not used for the adjustment because it was highly correlated with total cholesterol, which is problematic in regression models. GLMs were also adjusted for the time from baseline to the metabolic event for the primary outcome, and time from baseline to the end of follow-up and baseline TyG for the secondary outcome. These covariates were previously selected by a stepwise method (forward), according to that specific model’s lowest AKAike information criteria (AIC).

The statistical analysis was done with R statistical package (R version 4.2.0. R Foundation for Statistical Computing, Vienna, Austria). All *p*-values were corrected for multiple testing by using the Benjamini and Hochberg procedure (q-values), considering those values with *p*-value <0.05 (two-tailed) and q-value <0.20 as significant.

## Results

### Development of metabolic events

Characteristics of 80 persons with HIV/HCV coinfection according to the development of metabolic events are shown in Table 1. Overall, 78.8% were male, 64.1% were current smokers, and 45.6% and 81.2% had a prior history of alcohol intake and injection drug use, respectively. The median age was 51, and the BMI was 24.6 kg/m^2^. Regarding virological aspects, 74.7% were infected with HCV genotype 1; the CD4^+^ T cell count was 487.0 cells/mm^3^. With respect to HCV therapy, 40.0% of individuals received DAAs treatment, and 60.0% received IFN-based treatment (Table 1).

**TABLE 1:** Clinical, epidemiological, and virological characteristics of HIV/HCV-coinfected patients according to the development of metabolic events during the follow-up.

	All patients	Patients with metabolic events	Patients without metabolic events	*p*
No.	80	21 (26.3%)	59 (73.7%)	
Age (years)	51 (47–53)	50 (47–53)	51 (47–53)	0.831
Gender (male)	63 (78.8%)	17 (81.0%)	46 (78.0%)	0.999
BMI (kg/m^2^) (n = 77)	24.6 (21.9–28.7)	26.7 (24.5–31.4)	23.9 (21.4–27.7)	**0.008**
Smoker (n = 78)				0.149
Never	5 (6.4%)	0 (0.0%)	5 (8.8%)	
Previous (>6 months)	23 (29.5%)	9 (42.9%)	14 (24.6%)	
Current	50 (64.1%)	12 (57.1%)	38 (66.7%)	
Alcohol intake (>50g/day)				
(n = 79)				0.568
Never	40 (50.6%)	11 (52.4%)	29 (50.0%)	
Previous (>6 months)	36 (45.6%)	10 (47.6%)	26 (44.8%)	
Current	3 (3.8%)	0 (0.0%)	3 (5.2%)	
Intravenous drug user				0.776
Never	15 (18.8%)	3 (14.3%)	12 (20.3%)	
Previous (>6 months)	65 (81.2%)	18 (85.7%)	47 (79.7%)	
Current	0 (0%)	0 (0%)	0 (0%)	
Previous HCV therapy	48 (60.0%)	12 (57.1%)	36 (61.0%)	0.959
Lipid profile (mg/dL)				
Triglycerides (n = 78)	118.0 (87.8–173.5)	118.0 (93.8–177.0)	118.5 (86.3–166.8)	0.968
Total cholesterol (n = 76)	158.0 (135.5–180.0)	174.0 (153.5–193.5)	148.0 (127.0–172.0)	**0.008**
HDL cholesterol (n = 71)	38.0 (32.5–54.0)	41.0 (33.5–55.0)	38.0 (31.8–53.0)	0.603
LDL cholesterol (n = 71)	86.0 (64.0–107.0)	106.0 (89.0–121.0)	77.5 (59.0–103.0)	**0.003**
Liver markers				
LSM (kPa)	21.2 (13.5–34.9)	17.8 (13.5–27.7)	24.5 (13.7–35.0)	0.562
LSM (kPa) ≥ 12.5	64 (80.0%)	16 (76.2%)	48 (81.4%)	0.849
TyG (n = 78)	8.7 (8.3–9.0)	8.7 (8.6–9.1)	8.6 (8.2–9.0)	0.363
Ascites (n = 78)	8 (10.3%)	2 (9.5%)	6 (10.5%)	0.999
Bleeding esophageal varices (n = 79)	3 (3.8%)	2 (9.5%)	1 (1.7%)	0.349
Hepatic encephalopathy (n = 79)	4 (5.1%)	0 (0.0%)	4 (6.9%)	0.513
HCV markers				
HCV genotype (n = 79)				0.667
1	59 (74.7%)	15 (71.4%)	44 (75.8%)	
3	8 (10.1%)	2 (9.5%)	6 (10.3%)	
4	7 (8.9%)	3 (14.3%)	4 (6.9%)	
Others	5 (6.3%)	1 (4.8%)	4 (6.9%)	
Log_10_ HCV-RNA (IU/mL)				
(n = 79)	6.2 (5.7–6.6)	5.8 (5.1–6.2)	6.3 (5.9–6.7)	**0.008**
HCV-RNA > 850.000 IU/mL	52 (65.0%)	10 (47.6%)	42 (71.2%)	0.093
HCV therapy				0.641
IFN-based	48 (60.0%)	14 (66.7%)	34 (57.6%)	
DAAs	32 (40.0%)	7 (33.3%)	25 (42.4%)	
HIV markers				
Previous AIDS (n = 79)	2 (2.5%)	0 (0.0%)	2 (3.4%)	0.959
Nadir CD4+/mm^3^ (n = 78)	145 (83–257)	99 (48–242)	162 (95–260)	0.115
Nadir < 200 CD4+/mm^3^	50 (62.5%)	14 (66.7%)	36 (61.0%)	0.844
Baseline CD4+ T-cells/mm^3^	487 (293–713)	458 (350–662)	492 (267–718)	0.891
Baseline < 500 CD4+/mm^3^	41 (51.2%)	11 (52.4%)	30 (50.8%)	0.999
HIV antiretroviral therapy				
NRTI + NNRTI	20 (25.0%)	7 (33.3)	13 (22.0%)	0.060
NRTI + II	34 (42.5%)	4 (19.0)	30 (50.8%)	
NRTI + PI	9 (11.2%)	4 (19.0)	5 (8.5%)	
Others	17 (20.0%)	6 (28.6)	11 (18.6%)	

Statistics: The values are expressed as the absolute number (percentage) and median (interquartile range). *P*-values were calculated by the Chi-square test and the Mann-Whitney U test. Significant differences are shown in bold.

Abbreviations: BMI, body mass index; HCV, hepatitis C virus; HSI, hepatic steatosis index; LSM, liver stiffness measurement; kPa, kilopascal; TyG, triglyceride-glucose index; IU, international units; pegIFN, pegylated interferon; DAAs, direct-acting antivirals; HCV-RNA, viral load of hepatitis C; AIDS, acquired immune deficiency syndrome; NRTI, nucleoside analogue HIV reverse transcriptase inhibitor; NNRTI, non-nucleoside analogue HIV reverse transcriptase inhibitor; II, HIV integrase inhibitor; PI, HIV protease inhibitor; TyG, triglycerides and glucose index.

During the follow-up (median of 5 years after finishing HCV treatment), 21 (26%) patients developed metabolic events (T2DM and/or dyslipidemia). Of these, 7 (8.8%) and 15 (18.8%) patients developed T2DM and dyslipidemia, respectively, and only 1 (1.3%) patient developed both metabolic events. Similar characteristics were found between patients who developed metabolic events and those who did not, except for BMI (*p* = 0.008), total cholesterol (*p* = 0.008), LDL cholesterol (*p* = 0.003), and HCV viral load (*p* = 0.008) (Table 1).

**FIGURE 1:**
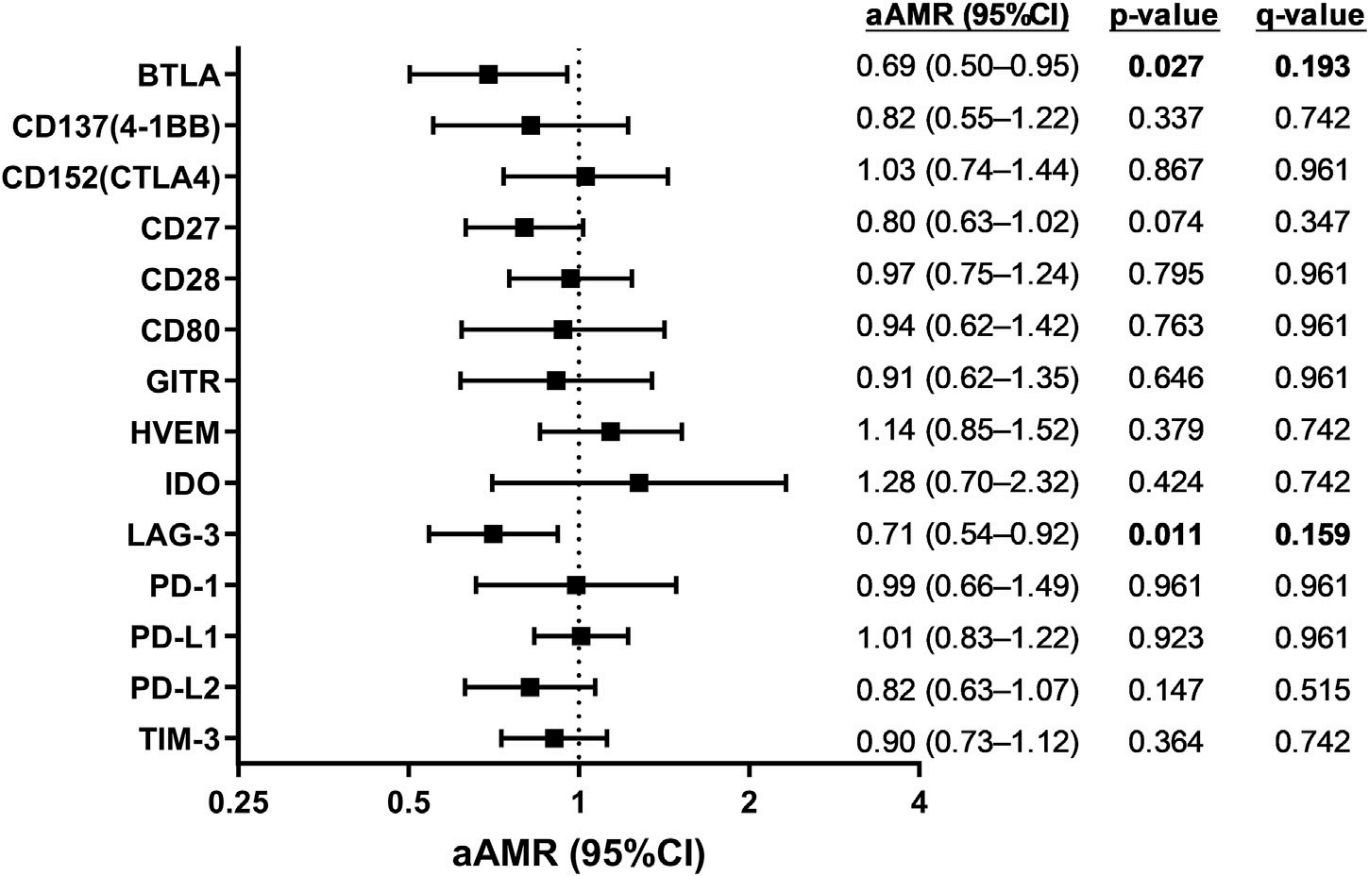
Association of plasma immune checkpoint proteins at baseline with the development of metabolic events during the follow-up in HIV/HCV-coinfected patients. Statistics: Data were calculated by Generalized Linear Models (GLM) with a gamma distribution (log-link) adjusted by age, gender, body mass index (BMI), total cholesterol, HCV viral load, and time from baseline to metabolic event time, previously selected by a stepwise method (see Results Section). Significant differences are shown in bold. Abbreviations: aAMR, adjusted AMR; 95%CI, 95% of confidence interval; BTLA, B, and T lymphocyte attenuator; CD, cluster of differentiation; GITR, glucocorticoid-induced TNFR-related; HVEM, herpesvirus entry mediator; IDO, indoleamine 2,3-dioxygenase; LAG-3, lymphocyte activation gene-3; PD-1, programmed cell death protein 1; PD-L1, programmed death-ligand 1; PD-L2, programmed death-ligand 2; TIM-3, T-cell immunoglobulin and mucin-domain containing-3.

Adjusted GLMs showed significant inverse associations (*p*-value <0.05 and q-value <0.20) of BTLA (aAMR = 0.69 (95%CI = 0.50–0.95)) and LAG-3 (aAMR = 0.71 (95%CI = 0.54–0.92)) with the development of metabolic events during the follow-up (Figure 1; Supplementary Table S1).

### Increased TyG values

**FIGURE 2:**
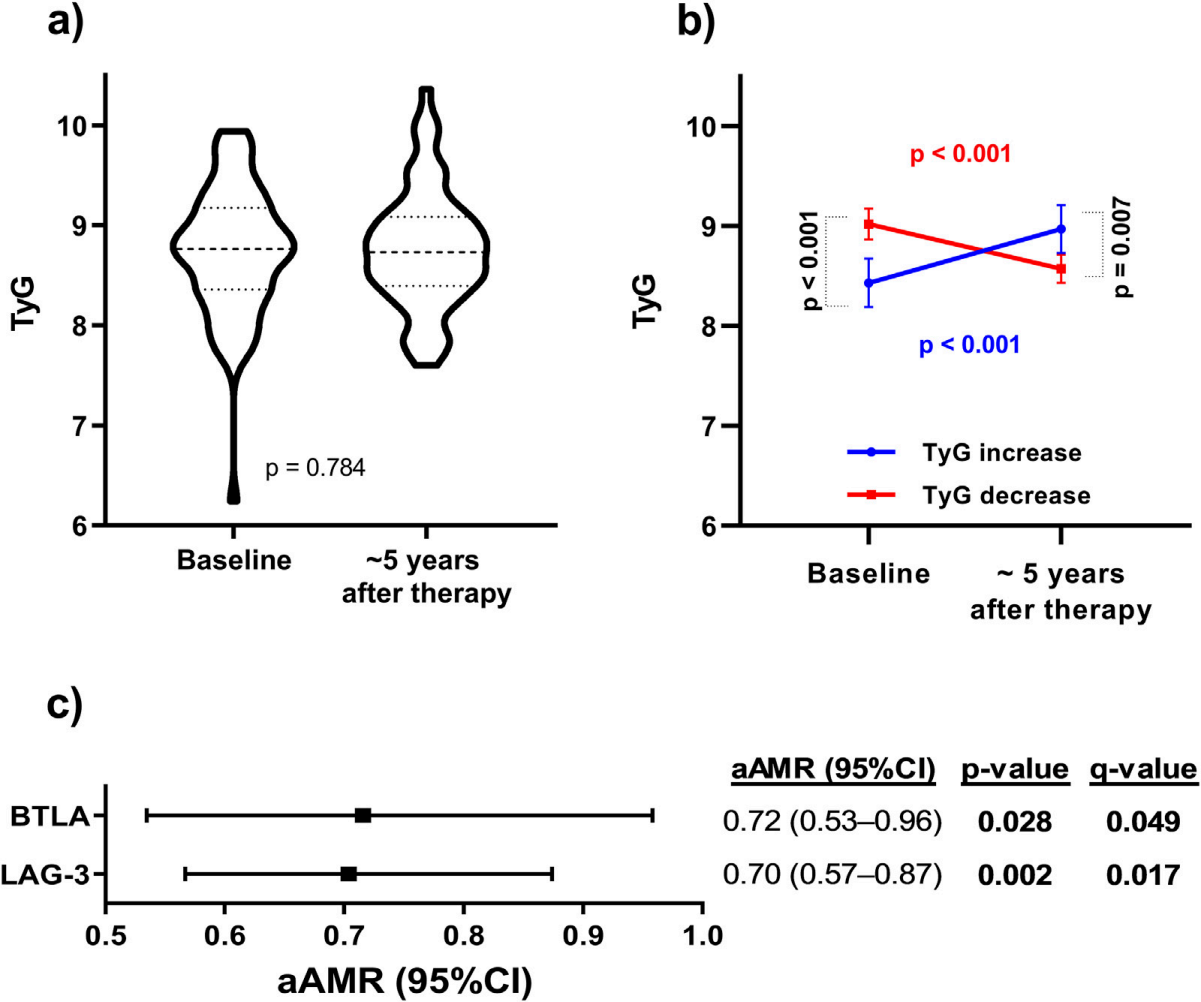
**(A)** Evolution of the TyG index related with metabolic syndrome from baseline to the end of follow-up (about 5 years after completing HCV treatment) in HIV/HCV-coinfected patients. Statistics: Data represent the medians and interquartile ranges for each time. **(B)** TyG index evolution for patients with TyG increase vs patients with TyG decrease during the follow-up. Statistics: Data represent the crude means and 95% confidence interval for each group of patients. *P*-values were calculated by the Mann-Whitney test for transversal analysis and the Wilcoxon test for longitudinal analysis between paired samples. **(C)** Association of plasma immune checkpoint proteins at baseline with the increase of TyG index to the end of follow-up in HIV/HCV-coinfected patients. Statistics: Data were calculated by Generalized Linear Models (GLM) with a gamma distribution (log-link) adjusted by age, gender, body mass index (BMI), total cholesterol, HCV viral load, triglyceride and glucose index (TyG), and time from baseline to the end of follow-up previously selected by a stepwise method (see Results Section). Abbreviations: TyG, triglyceride-glucose index; aAMR, adjusted AMR; 95%CI, 95% of confidence interval; BTLA, B, and T lymphocyte attenuator; CD, cluster of differentiation; GITR, glucocorticoid-induced TNFR-related; HVEM, herpesvirus entry mediator; IDO, indoleamine 2,3-dioxygenase; LAG-3, lymphocyte activation gene-3; PD-1, programmed cell death protein 1; PD-L1, programmed death-ligand 1; PD-L2, programmed death-ligand 2; TIM-3, T-cell immunoglobulin and mucin-domain containing-3.

Of the whole population, 69 patients had baseline and final TyG values; their characteristics are shown in Table 2. Overall, no significant differences in TyG values were found between the baseline and the end of the follow-up (Figure 2A), although 29 (46.0%) patients had a TyG increase (∆TyG>0) during the follow-up. Comparing patients with a TyG increase (∆TyG>0) versus patients with a TyG decrease (∆TyG<0), we found significant differences in TyG values at baseline (*p* < 0.001) and at the end of follow-up (*p* = 0.007) (Figure 2B). Significant differences (*p* < 0.001) were also observed between baseline and end of follow-up in both groups of patients.

**TABLE 2:** Clinical, epidemiological, and virological characteristics of HIV/HCV-coinfected patients according to triglyceride-glucose (TyG) index.

	All patients	Patients with TyG increase	Patients with TyG decrease	*p*
No.	69	33 (47.8%)	36 (52.2%)	
Age (years)	51 (48–53)	52 (51–54)	50 (48–53)	0.116
Gender (male)	56/69 (81.2%)	29/33 (87.9%)	27/36 (75%)	0.290
BMI (kg/m^2^)	24.7 (22.5–28.7)	25.5 (23.2–28.3)	24.6 (22.3–29)	0.485
Smoker				0.297
Never	4/68 (5.9%)	3/32 (9.4%)	1/36 (2.8%)	
Previous (>6 months)	20/68 (29.4%)	11/32 (34.4%)	9/36 (25%)	
Current	44/68 (64.7%)	18/32 (56.3%)	26/36 (72.2%)	
Alcohol intake (>50 g/day)				0.200
Never	34/69 (49.3%)	13/33 (39.4%)	21/36 (58.3%)	
Previous (>6 months)	32/69 (46.4%)	19/33 (57.6%)	13/36 (36.1%)	
Current	3/69 (4.3%)	1/33 (3%)	2/36 (5.6%)	
Intravenous drug user				0.291
Never	16/69 (23.2%)	10/33 (30.3%)	6/36 (16.7%)	
Previous (>6 months)	43/69 (76.8%)	23/33 (69.7%)	30/36 (83.3%)	
Current	0/69 (0%)	0/33 (0%)	0/36 (0%)	
Previous HCV therapy	43/69 (62.3%)	23/33 (69.7%)	20/36 (55.6%)	0.336
Lipid profile (mg/dL)				
Triglycerides	119 (93–178)	95 (73–118)	152.5 (118.8–213.8)	**<0.001**
Total cholesterol	154 (130–178)	142 (121–171)	160 (138.8–199.5)	**0.038**
HDL cholesterol	78 (59–105)	75.5 (60–101.5)	80 (63–117)	0.324
LDL cholesterol	39 (30–55)	41.50 (35.3–55.8)	38 (29.5–54)	0.392
Liver markers				
LSM	24.5 (14.3–34.3)	22 (16–34.3)	25 (14.2–34.1)	0.999
TyG	8.8 (8.4–9.2)	8.4 (8–8.8)	9 (8.7–9.3)	**<0.001**
Ascites	8/68 (11.8%)	6/33 (18.2%)	2/35 (5.7%)	0.223
Bleeding esophageal varices	2/69 (2.9%)	2/33 (6.1%)	0/36 (0%)	0.435
Hepatic encephalopathy	4/69 (5.8%)	3/33 (9.1%)	1/36 (2.8%)	0.545
HCV markers				
HCV genotype				0.744
1	48/65 (73.8%)	24/32 (75%)	24/33 (72.7%)	
3	9/65 (13.8%)	4/32 (12.5%)	5/33 (15.2%)	
4	7/65 (10.8%)	4/32 (12.5%)	3/33 (9.1%)	
Others	1/65 (1.5%)	0/32 (0%)	1/33 (3%)	
Log_10_ HCV-RNA (IU/mL)	6.2 (5.8–6.6)	6.1 (5.8–6.7)	6.3(5.8–6.5)	0.627
HCV-RNA >850.000 IU/mL	45/65 (65.2%)	20/32 (60.6%)	25/33 (69.4%)	0.605
HCV therapy				0.575
IFN-based	39/69 (56.6%)	17/33 (51.5%)	22/36 (61.1%)	
DAAs	30/69 (43.5%)	16/33 (48.5%)	14/36 (38.9%)	
HIV markers				
Previous AIDS	1/69 (1.4%)	0/33(0%)	1/36 (2.8%)	0.999
Nadir CD4+/mm^3^	130 (82–246)	150 (93–251)	117 (50–246)	0.232
Nadir <200 CD4+/mm^3^	45/68 (66.2%)	20/32 (62.5%)	25/36 (69.4%)	0.728
Baseline CD4^+^ T-cells/mm^3^	449 (280–723)	490 (348–721)	423 (245–752)	0.517
Baseline <500 CD4+/mm^3^	39/69 (56.5%)	17/33 (51.5%)	22/36 (61.1%)	0.575
HIV antiretroviral therapy				
NRTI + NNRTI	19/69 (27.5%)	10/33 (30.3%)	9/36 (25%)	0.263
NRTI + II	29/69 (42%)	16/33 (48.5%)	13/36 (36.1%)	
NRTI + PI	10/69 (14.5%)	2/33 (6.1%)	8/36 (22.2%)	
Others	11/69 (15.9%)	5/33 (15.1%)	6/36 (16.7%)	

Statistics: The values are expressed as the ratio (percentage) and median (interquartile range). *P*-values were calculated by the Chi-square test and the Mann-Whitney U test. Significant differences are shown in bold.

Abbreviations: BMI, body mass index; HCV, hepatitis C virus; HSI, hepatic steatosis index; LSM, liver stiffness measurement; kPa, kilopascal; TyG, triglyceride-glucose index; IU, international units; pegIFN, pegylated interferon; DAAs, direct-acting antivirals; HCV-RNA, viral load of hepatitis C; AIDS, acquired immune deficiency syndrome; NRTI, nucleoside analogue HIV, reverse transcriptase inhibitor; NNRTI, non-nucleoside analogue HIV, reverse transcriptase inhibitor; II, HIV, integrase inhibitor; PI, HIV, protease inhibitor; TyG, triglycerides and glucose index.

Significant inverse associations were found between baseline BTLA and LAG-3 levels and TyG increases (aAMR = 0.72 (95%CI = 0.53–0.96)) and (aAMR = 0.70 (95%CI = 0.57–0.87)), respectively; Figure 2C). Besides, other ICPs were also inversely associated with TyG increases: CD137(4-1BB) (aAMR = 0.62 (95%CI = 0.44–0.88)), CD152(CTLA4) (aAMR = 0.61 (95%CI = 0.45–0.84)), CD27 (aAMR = 0.80 (95%CI = 0.67–0.95)), CD28 (aAMR = 0.72 (95%CI = 0.58–0.89)), GITR (aAMR = 0.70 (95%CI = 0.50–0.98)), HVEM (aAMR = 0.72 (95%CI = 0.57–0.91)), PD-1 (aAMR = 0.66 (95%CI = 0.48–0.92)), PD-L1 (aAMR = 0.85 (95%CI = 0.73–0.99)), and TIM-3 (aAMR = 0.82 (95%CI = 0.68–0.99)) (Supplementary Table S2).

## Discussion

Our analysis revealed that patients with low baseline levels of BTLA and LAG-3 experienced a significantly higher incidence of metabolic disorders within 5 years post-HCV therapy. Similarly, several plasma ICPs (including BTLA and LAG-3) were inversely associated with a long-term increase in TyG values, a simple, non-invasive indicator of metabolic syndrome (Song et al., 2022), supporting their association with the development of metabolic events.

Metabolic disorders are quite prevalent in HCV-infected patients (Collins et al., 2019); however, the long-term impact of HCV clearance on metabolic diseases has been scarcely studied so far. To date, HCV infection has been inversely associated with dyslipidemia, and a rebound effect of serum lipid levels has been observed after SVR induced by both interferon-based therapy and DAAs (Wang et al., 2020). Regarding the baseline, plasma triglycerides level is reduced, but the cholesterol and low-density lipoprotein cholesterol (LDL-C) levels increase gradually after HCV treatment (Carrero et al., 2020). Likewise, although it has been described that reaching SVR has a protective effect against the incidence of new-onset T2DM (Berenguer et al., 2017), the presence of T2DM has been described as a significant risk factor for hepatocellular carcinoma after SVR (Luna-Cuadros et al., 2022). However, this issue requires further investigation. Most studies have focused on patients with prior metabolic syndrome and short-term follow-up after treatment (Collins et al., 2019; Wang et al., 2020). In contrast, our work is the first to conduct a long-term follow-up study including pre-treated patients who will develop metabolic disorders – or who are at risk of developing them – after achieving SVR.

In the current study, more than a quarter of patients who achieved SVR developed metabolic events during follow-up. Since it is crucial to monitor metabolic factors and take timely measures to prevent or treat metabolic disorders in HCV-clearing patients, it is highly desirable to identify predictive biomarkers that predict metabolic events, identifying patients who could benefit from closer follow-up after HCV eradication.

As illustrated in Figure 1, there was a notable inverse correlation between baseline BTLA and LAG-3 levels and metabolic event occurrence. BTLA exerts inhibitory effects, reducing T-cell activation and decreasing cytokine production, cell proliferation, and cell cycle progression (Ning et al., 2021). LAG-3 also negatively modulates T cell function by ligation with the major histocompatibility complex (MHC) class II expressed on the surface of antigen-presenting cells (Burnell et al., 2022). There is not much direct evidence on the relationship between BTLA and metabolic alterations unrelated to autoimmune diseases, unlike LAG-3, which does appear to be associated. Reduced LAG-3 levels (by global knockout and/or antibody blockade) in non-obese diabetic animals accelerate metabolic events (Previte et al., 2019). Decreased levels of LAG3 soluble have been associated with BMI and diabetes mellitus (Xiong et al., 2022). High plasma LAG-3 level has been positively associated with high IL-10 levels, an anti-inflammatory cytokine, suggesting that low LAG-3 levels may promote pro-inflammatory effects (Golden et al., 2016). T2DM in patients with metastatic melanoma appears to be associated with lower LAG-3 levels (Mallardo et al., 2022).

In our study, we also found significant inverse associations of ICPs before HCV therapy with the long-term increase in TyG values during the follow-up, which supports the association of reduced BTLA and LAG-3 levels with the development of metabolic alterations. Among them, HVEM binds to BTLA and provides inhibitory signals in activated T- and B- cells (Ning et al., 2021). CD152(CTLA4), a T-cell receptor that recognizes CD80/CD86 is a critical down-regulator of T-cells (Stirling et al., 2022). TIM-3, a T-cell receptor that inhibits T-cell activity, induces the expansion of myeloid-derived suppressor cells and regulates self-tolerance (Wang et al., 2022). PD-1 binds to PD-L1 and PDL-2, inhibiting immune responses and stimulating self-tolerance by controlling T-cell activation and proliferation (Singh et al., 2021). Consistent with our results, previous studies described that animal and cellular models lacking PD-L1 and PD-1 had an increase in atherosclerosis and cholesterol levels (Gotsman et al., 2007; Bu et al., 2011).

Among other functions, ICPs modify the cellular metabolism (Stirling et al., 2022), supporting the possible role of these biomarkers in the pathophysiology of metabolic disorders in persons with HIV/HCV coinfection who cleared HCV infection. In this regard, it is well-known that immune checkpoint inhibitor (ICIs) therapies recently used for the treatment of cancer (including anti-CD152(CTLA4), anti-PD-1/PD-L1, anti-BTLA, or anti-LAG-3) (Chen et al., 2019; Huo et al., 2022), enhance host immunity, promoting the inflammatory response, and leading to adverse immune-related metabolic disorders (Leiter et al., 2021). Likewise, an increased incidence of cardiovascular immune-related adverse events has also been associated with ICIs due to dyslipidemia and atherosclerosis (Dolladille et al., 2021), which are frequent sources of acute and persistent morbidity that can be fatal (Wright et al., 2021). Several studies have demonstrated the association between ICIs and the development of pancreatic β cell failure leading to insulin deficiency, hyperglycemia, and type 1 diabetes (Kotwal et al., 2019), as well as increased atherosclerotic burden (Ley et al., 2017). ICIs can also worsen glycemic control in pre-existing T2DM (Kotwal et al., 2019). This evidence suggests that lower plasma levels of ICPs analyzed here in persons with HIV/HCV coinfection might allow upregulation of host immunity, like those in oncology patients treated with ICIs, increasing the risk of metabolic disorders.

Finally, it is essential to note that these ICPs are expressed not only in T-cells, but also in a wide variety of immune cells and other locations such as dendritic cells, hepatocytes, and pancreatic islet cells, among others. Soluble forms of these ICPs are also generated, and although their activities remain unclear, they also appear to modulate immune responses (Gu et al., 2018). Thus, a complex interaction network of these ICPs with each other – including soluble forms – and with other molecules of the immune system makes their understanding a challenge (Gaikwad et al., 2022), being necessary for additional studies to corroborate our findings.

The following limitations should be considered for a correct interpretation of the study. First, the limited sample size could have restricted the detection of positive associations with other ICPs. In addition, the modest sample size may also increase the false positive rate, but our positive findings were FDR-corrected, lending robustness to our results. Second, the modest sample size also did not allow for studying the association between diabetes and dyslipidemia separately, which would have provided additional information. Third, the study design was retrospective and may have introduced biases, such as different HCV therapies for treating patients (IFN and DAA-based treatment). However, no significant differences were found between groups of patients. Fourth, comparison control groups were not available, such as people with untreated HIV/HCV c-infection or those treated but not achieving SVR. Fifth, association analysis could not be performed separately by sex because of the limited sample size. Further studies are needed to evaluate the utility of these biomarker profiles for identifying persons with HIV/HCV coinfection who need closer monitoring after successful HCV therapy. Another limitation was that diabetes and hyperlipidemia were defined using single laboratory measurements, which is less rigorous than requiring abnormal laboratory values in serial measurements (at least two), potentially leading to misclassification.

In conclusion, lower baseline plasma levels of BTLA and LAG-3 before HCV therapy were associated with the development of metabolic events and long-term increases in TyG values during the first 5 years after successful completion of anti-HCV therapy, supporting the possible role of these molecules in the pathophysiology of metabolic disturbances in persons with HIV/HCV coinfection.

## Data Availability

The raw data supporting the conclusions of this article will be made available by the authors, without undue reservation.

## Group members of: The marathon study group

Hospital General Universitario Gregorio Marañón, Madrid: T Aldamiz-Echevarría, J Bermejo, J Berenguer, J. M. Bellón, A Carrero, S Carretero, C Díez, C Fanciulli, I Gutiérrez, J. C. López, P Miralles, F Parras, B Padilla, L Pérez-Latorre, M Ramírez, F Tejerina. Hospital Universitario La Paz, Madrid: J. R. Arribas, F. Arnalich, I. Bernardino, M. Díaz, J. González-García, V. Hontañón, M. L. Montes, J. F. Pascual, J. M. Peña, F. Zamora. Hospital Universitari Vall d’Hebron, Barcelona: M. Crespo, M. Pérez, E. Ribera, E. Van den Eynde. Hospital Universitario Príncipe de Asturias, Alcalá de Henares: A. Arranz, E Casas, J. De Miguel, S. Schroeder, J Sanz. Hospital Donostia, San Sebastián: M. J. Bustinduy, J. A. Iribarren, F. Rodríguez-Arrondo, M. A. Von-Wichmann. Hospital Universitario de La Princesa, Madrid: I. Santos, J. Sanz. Hospital Clínico San Carlos, Madrid: M. J. Téllez, J. Vergas. Hospital Clínico Universitario, Valencia: A. Ferrer, M. J. Galindo. Hospital Universitario Ramón y Cajal Madrid: J. L. Casado, F. Dronda, A. Moreno, S. Moreno, M. J. Pérez-Elías, C. Quereda, M. A. Sanfrutos. Hospital General Universitario, Valencia: E. Ortega, L. Ortiz. Fundación SEIMC-GESIDA, Madrid: E. Aznar, P. Crespo, H. Esteban, M. Yllescas.

## Glossary

ALT: alanine aminotransferase
AMR: arithmetic mean ratio
AST: aspartate aminotransferase
BMI: body mass index
BTLA: B and T lymphocyte attenuator
ART: antiretroviral therapy
CD: cluster of differentiation
CI: confidence interval
DAAs: direct-acting antivirals
FDR: false discovery rate
GITR: glucocorticoid-induced TNFR-related
GLM: Generalized Linear Models
HBV: hepatitis B virus
HCC: hepatocellular carcinoma
HCV: hepatitis C virus
HIV: human immunodeficiency virus
HSI: hepatic steatosis index
HVEM: herpesvirus entry mediator
IPCs: immune checkpoint proteins
IDO: indoleamine 2,3-dioxygenase
IL-1RA: interleukin 1 receptor antagonist
IL-18: interleukin 18
IL-8: interleukin 8
IP-10: human interferon-inducible protein 10
IQR: interquartile range
LAG-3: lymphocyte activation gene-3
LSM: liver stiffness measurement
MCP-1: monocyte chemoattractant protein-1
NAFLD: non-alcoholic fatty liver disease
PD-1: programmed cell death protein 1
PD-L1: programmed death-ligand 1
PD-L2: programmed death-ligand 2
pegIFN: pegylated interferon
SVR: sustained virologic response
TIM-3: T-cell immunoglobulin and mucin-domain containing-3
TNF-RI: tumor necrosis factor receptor-1
TyG: Triglycerides and glucose index
