# Supplementary material for: Low plasma levels of BTLA and LAG-3 before HCV therapy are associated with metabolic disorders after HCV eradication in persons with HIV/HCV coinfection: a retrospective study
Source: Front Pharmacol. 2024 Oct 28;15:1341612. doi: 10.3389/fphar.2024.1341612 (PMC11551606; doi:10.3389/fphar.2024.1341612)
Supplement: Supplementary file 1 [file Table1.docx]

**Supplementary Table 1**. Association of plasma immune checkpoint proteins at baseline with the development of metabolic events during the follow-up time in HIV/HCV-coinfected patients.

|  | **Un-adjusted** | | | **Adjusted** | | |
| --- | --- | --- | --- | --- | --- | --- |
| **Marker** | **AMR (95%CI)** | ***p*-value** | ***q*-value** | **aAMR (95%CI)** | ***p*-value** | ***q*-value** |
| BTLA | 0.72 (0.53–0.97) | **0.033** | 0.229 | 0.69 (0.50–0.95) | **0.027** | **0.193** |
| CD137(4-1BB) | 0.82 (0.56–1.19) | 0.292 | 0.689 | 0.82 (0.55–1.22) | 0.337 | 0.742 |
| CD152(CTLA4) | 1.04 (0.73–1.48) | 0.826 | 0.895 | 1.03 (0.74–1.44) | 0.867 | 0.961 |
| CD27 | 0.91 (0.74–1.12) | 0.394 | 0.787 | 0.80 (0.63–1.02) | 0.074 | 0.347 |
| CD28 | 1.00 (0.79–1.26) | 0.971 | 0.971 | 0.97 (0.75–1.24) | 0.795 | 0.961 |
| CD80 | 0.76 (0.53–1.09) | 0.135 | 0.472 | 0.94 (0.62–1.42) | 0.763 | 0.961 |
| GITR | 0.91 (0.64–1.30) | 0.619 | 0.787 | 0.91 (0.62–1.35) | 0.646 | 0.961 |
| HVEM | 1.08 (0.85–1.39) | 0.527 | 0.787 | 1.14 (0.85–1.52) | 0.379 | 0.742 |
| IDO | 1.06 (0.60–1.87) | 0.831 | 0.895 | 1.28 (0.70–2.32) | 0.424 | 0.742 |
| LAG-3 | 0.74 (0.58–0.94) | **0.014** | 0.203 | 0.71 (0.54–0.92) | **0.011** | **0.159** |
| PD-1 | 0.82 (0.57–1.18) | 0.295 | 0.689 | 0.99 (0.66–1.49) | 0.961 | 0.961 |
| PD-L1 | 0.95 (0.80–1.13) | 0.580 | 0.787 | 1.01 (0.83–1.22) | 0.923 | 0.961 |
| PD-L2 | 0.81 (0.64–1.04) | 0.100 | 0.469 | 0.82 (0.63–1.07) | 0.147 | 0.515 |
| TIM-3 | 0.94 (0.76–1.15) | 0.536 | 0.787 | 0.90 (0.73–1.12) | 0.364 | 0.742 |

**Statistics:** Data were calculated by Generalized Linear Models (GLM) with a gamma distribution (log-link). Multivariable models were adjusted by age, gender, body mass index (BMI), HCV viral load, and time from baseline to metabolic event time, previously selected by a stepwise method (forward) (see **Results Section**). The q-values represent p-values corrected for multiple testing using the False Discovery Rate (FDR). Statistically significant differences are shown in bold.

**Abbreviations**: AMR, arithmetic mean ratio; aAMR, adjusted AMR; 95%CI, 95% of confidence interval; p, level of significance; q, corrected level of significance; BTLA, B and T lymphocyte attenuator; CD, cluster of differentiation; 137GITR, glucocorticoid-induced TNFR-related; HVEM, herpesvirus entry mediator; IDO, indoleamine 2,3-dioxygenase; LAG-3, lymphocyte activation gene-3; PD-1, programmed cell death protein 1; PD-L1, programmed death-ligand 1; PD-L2, programmed death-ligand 2; TIM-3, T-cell immunoglobulin and mucin-domain containing-3.

**Supplementary Table 2**. Association of plasma immune checkpoint proteins at baseline with increased triglyceride and glucose index (TyG) at the end of follow-up in HIV/HCV-coinfected patients.

|  | **Un-adjusted** | | | **Adjusted** | | |
| --- | --- | --- | --- | --- | --- | --- |
| **Marker** | **AMR (95%CI)** | ***p*-value** | ***q*-value** | **aAMR (95%CI)** | ***p*-value** | ***q*-value** |
| BTLA | 0.69 (0.51–0.94) | **0.021** | **0.036** | 0.72 (0.53–0.96) | **0.028** | **0.049** |
| CD137(4-1BB) | 0.62 (0.43–0.89) | **0.011** | **0.031** | 0.62 (0.44–0.88) | **0.010** | **0.029** |
| CD152(CTLA4) | 0.68 (0.49–0.95) | **0.026** | **0.037** | 0.61 (0.45–0.84) | **0.004** | **0.017** |
| CD27 | 0.81 (0.68–0.97) | **0.023** | **0.036** | 0.80 (0.67–0.95) | **0.014** | **0.032** |
| CD28 | 0.75 (0.60–0.93) | **0.010** | **0.031** | 0.72 (0.58–0.89) | **0.003** | **0.017** |
| CD80 | 0.70 (0.49–1.00) | 0.053 | 0.062 | 0.85 (0.57–1.27) | 0.441 | 0.475 |
| GITR | 0.67 (0.49–0.93) | **0.021** | **0.036** | 0.70 (0.50–0.98) | **0.041** | **0.057** |
| HVEM | 0.72 (0.57–0.90) | **0.006** | **0.030** | 0.72 (0.57–0.91) | **0.008** | **0.028** |
| IDO | 0.93 (0.53–1.62) | 0.800 | 0.800 | 1.11 (0.62–1.99) | 0.729 | 0.729 |
| LAG-3 | 0.71 (0.56–0.90) | **0.006** | **0.030** | 0.70 (0.57–0.87) | **0.002** | **0.017** |
| PD-1 | 0.62 (0.45–0.87) | **0.006** | **0.030** | 0.66 (0.48–0.92) | **0.017** | **0.034** |
| PD-L1 | 0.87 (0.74–1.03) | 0.101 | 0.109 | 0.85 (0.73–0.99) | **0.049** | **0.063** |
| PD-L2 | 0.78 (0.62–0.98) | **0.034** | **0.043** | 0.84 (0.68–1.04) | 0.109 | 0.139 |
| TIM-3 | 0.80 (0.67–0.96) | **0.019** | **0.036** | 0.82 (0.68–0.99) | **0.038** | **0.057** |

**Statistics:** Data were calculated by Generalized Linear Models (GLM) with a gamma distribution (log-link). Multivariable models were adjusted by age, gender, body mass index (BMI), total cholesterol, HCV viral load, triglyceride and glucose index (TyG), and time from baseline to the end of follow-up previously selected by a stepwise method (forward) (see **Results Section**). The q-values represent p-values corrected for multiple testing using the False Discovery Rate (FDR). Statistically significant differences are shown in bold.

**Abbreviations**: AMR, arithmetic mean ratio; aAMR, adjusted AMR; 95%CI, 95% of confidence interval; p, level of significance; q, corrected level of significance; BTLA, B and T lymphocyte attenuator; CD, cluster of differentiation; GITR, glucocorticoid-induced TNFR-related; HVEM, herpesvirus entry mediator; IDO, indoleamine 2,3-dioxygenase; LAG-3, lymphocyte activation gene-3; PD-1, programmed cell death protein 1; PD-L1, programmed death-ligand 1; PD-L2, programmed death-ligand 2; TIM-3, T-cell immunoglobulin and mucin-domain containing-3.
